# A novel radiological method to evaluate the posterior tympanotomy depth for cochlear implantation: our experience in 257 patients

**DOI:** 10.1007/s00405-022-07334-6

**Published:** 2022-03-26

**Authors:** Mahmoud Mandour, Haitham H. Elfarargy, Rasha Lotfy, Mohamed N. Elsheikh, Maurizio Barbara, Saad Elzayat

**Affiliations:** 1 Otolaryngology Department, Tanta University, Tanta, Egypt; 2 Otolaryngology Department, Kafrelsheikh University, Elgeeish Street, Kafrelsheikh, 33511 Egypt; 3 Radiology Department, Tanta University, Tanta, Egypt; 4 Otolaryngology Department, Sapienza University of Rome, Rome, Italy

**Keywords:** Cochlear implantation, Radiology, Posterior tympanotomy depth

## Abstract

**Purpose:**

This study aimed to validate our novel proposed radiological evaluation of the posterior tympanotomy (PT) depth. This dimension represents the bone of the facial recess needed to be drilled to get access into the middle ear during cochlear implantation.

**Methods:**

It was a retrospective observational study that included 257 patients who underwent cochlear implantation from July 2018 to April 2021 in tertiary referral institutions. Two physicians evaluated the preoperative HRCT to measure the PT depth in the oblique para-sagittal cut. On the other hand, two other physicians evaluated the unedited surgical videos to judge the PT depth and classified it into an ordinary PT or deep PT. Then, the preoperative radiological measurements were correlated with the intraoperative findings.

**Results:**

The radiological PT depth ranged from 2.5 to 5.4 mm with a mean of 3.91 ± 0.886. Sixty-six patients had ordinary PT, and 191 patients had deep PT. Spearman’s correlation coefficient revealed a strong correlation between the preoperative radiological PT depth measurements and the intraoperative PT depth judgments (*p* value < 0.0001).

**Conclusions:**

We created a novel radiological method to measure the posterior tympanotomy depth. This method was valid, reproducible, and reliable in the preoperative radiological evaluation of the PT depth with high sensitivity (91.71%), specificity (90.62%), and accuracy (91.44%). We also found a significant impact of the PT depth on the PT difficulty during cochlear implantation.

## Introduction

The facial recess is located in the posterior wall of the middle ear. It is defined as the triangular region between the vertical segment of the facial nerve medially, the chorda tympani nerve laterally, and the fossa incudis superiorly. It is a significant landmark as it provides a route for a posterior tympanotomy surgical approach [1–3].

Jansen was the first to describe the posterior tymapanotomy approach in 1958 [4]. This technique is done by drilling the bony facial recess to expose the mesotympanic part of the middle ear. The posterior tympanaotomy approach has many otological applications as electrode insertion during cochlear implantation, ossiculoplasty, facial nerve decompression, middle ear cholesteatoma removal, middle ear implant placement, and lateral temporal bone dissection [5].

The frequent use of posterior tympanotomy in ear surgeries and its relation to critical structures as the facial nerve necessitates an accurate analysis of the facial recess. Therefore, the facial recess has been an interesting point for many anatomical previous studies [6, 7]. Also, many researchers used HRCT to analyze the FR radiologically. These researchers tried to help the surgeon make a safe, optimal posterior tympanotomy [8].

Most researchers concentrated mainly on the width and length of the facial recess, ignoring the PT depth [9]. This study tried to find an appropriate radiological method to measure the PT depth. We also attempted to validate this method by correlating it with the intraoperative findings.

## Patients and methods

### Ethics

We initially obtained the institutional review board approval of Kafrelsheikh University to conduct this study. The included patients’ guardians signed an informed agreement on using the data of their children in our research. All included procedures were performed according to the Declaration of Helsinki [10].

### Study design

It was a retrospective observational case-series study.

### Setting and duration

Senior CI surgeons (M. Mandour and S. Elzayat) performed the cochlear implant surgeries at tertiary referral institutions of cochlear implantation through the national cochlear implant program from July 2018 to April 2021.

### Subjects

We included 257 pediatric patients who underwent CI surgery through the posterior tympanotomy (PT) approach. We only had both preoperative HRCT scans, and their unedited surgical video record was present. We excluded cases with previous ear surgeries, middle ear inflammation (cholesteatoma, otitis media with effusion), other approaches for CI, congenital cochlea-vestibular anomalies, external auditory canal (EAC) anomalies, preoperative facial paralysis, and revision CI. So, we excluded twenty-three cases.

### CT protocol

**Fig. 1:**
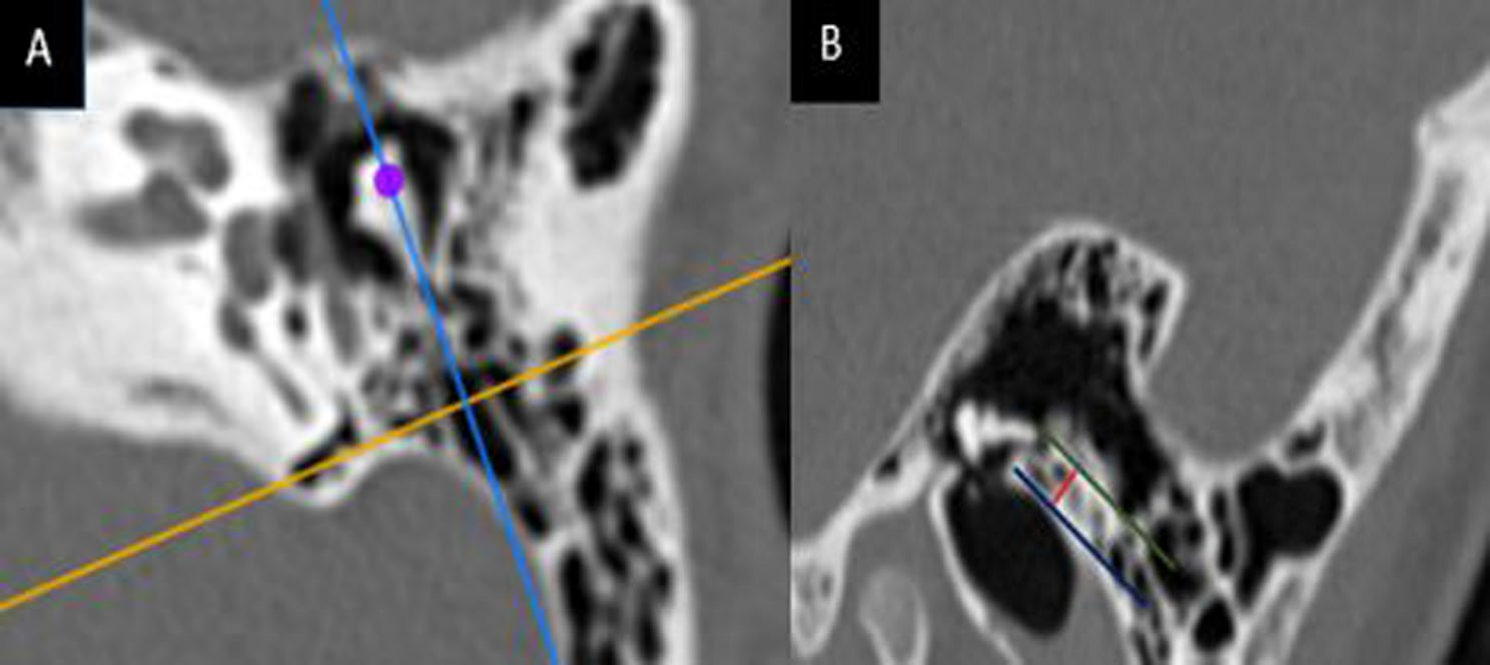
The left coronal view showed the axes of scrolling to get the oblique para-sagittal view. **A** Left oblique para-sagittal view. The green line passed tangentially to the tip of the incus short process. The blue line passed tangentially to the posterior wall of the EAC. The red line represented the distance between the two last lines. In this case, PT depth was 2.9 mm and was classified as an ordinary depth PT

Radiological imaging was performed using an HRCT machine. The CT scan data were acquired at 120 kV, 200 mA, and the imaging matrix of 512 × 512. The axial cuts were obtained parallel to the orbito-meatal baseline and viewed in the standard bone window settings. Coronal cuts were made in a plane perpendicular to axial images at 0.6–0.5 mm intervals. The technologist scrolled through the coronal plane to get the transverse oblique coronal long axis (oblique para-sagittal) (Fig. 1).

### CT reviewing

CI surgeon and temporal bone radiologist reviewed the preoperative HRCT images. They independently measured the PT depth. In the oblique parasagittal reconstructed plane along the short process of the incus, a line was drawn tangential to the medial extension of the posterosuperior meatal wall. Another line was drawn from the tip of the short process parallel to the first line. The distance between the two lines represented the PT depth. They also classified the posterior tympanotomy radiologically into:

- Ordinary PT: if the PT depth was equal to or less than 3.1 mm.
- Deep PT: if the PT depth was more than 3.1 mm.

### Video reviewing

Two CI surgeons, blind to the before-mentioned radiological assessments, independently reviewed the patients’ unedited video records. They judged the intraoperative posterior tympanotomy depth according to the amount of the compact bone needed to be drilled starting from a virtual coronal plane (posteroanterior plane) passing through the incus short process tip to gain access into the middle ear through the facial recess (FR thickness). According to this qualitative judgment, PT was classified into:

- Ordinary PT: if an average amount of bone was drilled (reasonable FR thickness).
- Deep PT: if there was a need to remove an extensive amount of bone to reach the middle ear (thick FR).

The two video reviewers also judged the PT difficulty. This judgment depended on the need for extra surgical steps to reach an accessible round window (RW) for atraumatic electrode insertion. These steps included the reallocation of the microscope, extreme thinning of the meatal wall, uncapping the mastoid portion of the facial nerve, sacrificing the chorda tympani nerve, or removing the incus buttress. They classified the PT according to the need of these extra steps into:

- Straightforward PT: there was not a need for extra steps to get an accessible RW.
- Challenging PT: If extra steps were needed to get an accessible RW.

### Outcome measures

We correlated the preoperative radiological PT depth measurement and classification with the intraoperative findings. We assessed the ability of our radiological technique to predict the intraoperative PT depth. We also evaluated the impact of the PT depth on the PT difficulty.

### Statistical analysis

Statistical analysis was done using SPSS v22 (IBM^©^ Inc., Chicago, IL, USA). Numerical variables were presented as mean and standard deviation (SD). Categorical variables were presented as frequency and percentage (%). We used Mann–Whitney to compare both groups. *p* value < 0.05 was considered significant. We used Spearman’s correlation coefficient to detect the relationship between the radiological PT depth measurement and the intraoperative depth. We used the intra-class correlation coefficient test to assess the inter-observer variability. We held the receiver operating characteristic (ROC) curve between the radiological PT depth measurement and intraoperative PT depth classification.

## Results

### PT depth judgement in the surgical videos

Our study included 257 patients. According to the intraoperative PT depth in the surgical videos, these patients were divided into two groups. The first group included 66 patients whose intraoperative PT depth was ordinary (Group A). On the other hand, group B included 191 patients whose intraoperative PT was deep. Both video reviewers' intraoperative PT depth judgments were strongly correlated as the intra-class correlation coefficient was 0.908 (Table 2).

### Demographic results

We included 147 males and 110 females. The aged ranged from 2.1 years to 7.2 years with a mean of 4.64 ± 1.198. Two hundred twenty-two cases were implanted on the right side. At the same time, 35 patients had their CI on the left side. The patients’ age, sex, and operation site did not show a statistically significant difference between both groups (*p* value was > 0.05) (Table 1).

**Table 1:** The results of all included patients

Age		Minimum (years)	2.1
		Maximum (years)	7.2
		Mean ± SD (years)	4.64 ± 1.198
Sex		Males	147 (57.2%)
		Females	110 (48.8%)
Side of operated ear		Right	222 (86.4%)
		Left	35 (13.6%)
Preoperative CT PT depth	Measurement	Minimum (mm)	2.5
		Maximum (mm)	5.4
		Mean ± SD (mm)	3.91 ± 0.886
		ICC	0.987
	Grading	Ordinary depth	76 (29.6%)
		Deep	181 (70.4%)
		ICC	0.809
Intraoperative PT depth		Ordinary depth	66 (25.7%)
		Deep	191 (74.3%)
		ICC	0.908
Intraoperative PT difficulty		Straightforward	181 (70.4%)
		Challenging	76 (29.6%)
		ICC	0.885

*SD* standard deviation, *PT* posterior tympanotomy, *ICC* intra-class correlation coefficient

### PT depth measurements in the preoperative CT

The preoperative radiological PT depth of all patients ranged from 2.5 to 5.4 mm with a mean of 3.91 ± 0.886 mm (Table 1). In group A, the radiological PT depth ranged from 2.5 to 3.4 mm with a mean of 2.85 ± 0.2 mm. While in group B, it went from 2.8 to 5.4 mm with a mean of 4.27 ± 0.722 mm. Both groups’ preoperative radiological PT depth measurement differed significantly as the *p* value was < 0.0001. The intra-class correlation coefficient between the PT depth measurements of both CT reviewers was 0.987. This indicated a vital harmony between both measures. It also revealed the reliability of our radiological measurement method of PT depth in the HRCT (Table 2).

**Table 2:** The results of both groups

			Group A	Group B	*p* value
			(*N* = 66)	(*N* = 191)	
		Minimum (years)	2.1	2.1	
Age		Maximum (years)	7.1	7.2	0.388
		Mean ± SD (years)	4.75±1.2	4.6± 1.19	
Sex		Males	38 (57.6%)	109 (57.1%)	0.943
		Females	28 (42.4%)	82 (42.9%)	
Side of operated ear		Right	55 (83.3%)	167 (87.4%)	0.403
		Left	11 (16.7%)	24 (12.6%)	
		Minimum (mm)	2.5	2.8	
	Measurement	Maximum (mm)	3.4	5.4	< 0.0001*
Preoperative CT PT depth		Mean ± SD (mm)	2.85± 0.2	4.27±0.722	
	Grading	Ordinary depth	60 (90.9%)	16 (8.4%)	< 0.0001*
		Deep	6 (9.1%)	175 (91.6%)	
Intraoperative PT difficulty		Straightforward	60 (90.9%)	121 (63.4%)	< 0.0001*

Group A: ordinary PT depth in the surgical videos, Group B: deep PT in the surgical videos

*SD* standard deviation, *PT* posterior tympanotomy

*Statistically significant as the *p* value < 0.05)

### The PT depth judgement in the preoperative CT

The preoperative CT reviewers categorized the PT of all included patients radiologically into 76 patients with an ordinary PT depth and 181 deep posterior tympanotomies. The intra-class correlation coefficient was 0.809, which indicated a strong correlation between both CT reviewers’ judgments (Table 1).

According to the CT evaluation, 60 patients had a radiological ordinary PT depth and 6 patients were classified as deep PT in the preoperative HRCT of group A. On the other side, 16 patients were radiologically classified as ordinary PT depth, and 175 patients as deep PT in the preoperative HRCT of group B. Both groups’ preoperative PT depth type categorization showed a statistically significant difference as the *p* value was < 0.0001 (Table 2).

### The PT difficulty judgment in the surgical videos

According to the posterior tympanotomy difficulty in the surgical videos of all patients, the video reviewers found that PT was straightforward in 181 cases. In contrast, it was challenging in 76 patients. PT difficulty results of both video reviewers were correlated as the intra-class correlation coefficient was 0.885 (Table 1).

According to the posterior tympanotomy difficulty in the surgical videos, PT was straightforward in 60 cases and challenging in 6 cases of group A. On the other hand, PT was straightforward in 121 patients and challenging in 70 cases of group B. The PT difficulty showed a statistically significant difference between both groups as the *p* value was < 0.0001 (Table 2).

### Bivariate analysis

The intraoperative PT depth type was strongly related to the radiological measurement of PT depth in the preoperative HRCT as Spearman's correlation coefficient was 0.716 and *p* value was < 0.0001. Additionally, Spearman’s correlation coefficient revealed a significant impact of PT depth on the PT difficulty (*p* value was < 0.0001). The positivity of Spearman’s correlation coefficient (0.0409) between the radiological PT depth measurement and intraoperative PT difficulty indicated a positive relationship between them; when the depth increased, the difficulty increased and the reverse.

### Sensitivity of the radiological PT depth measurement in the CT

**Fig. 2:**
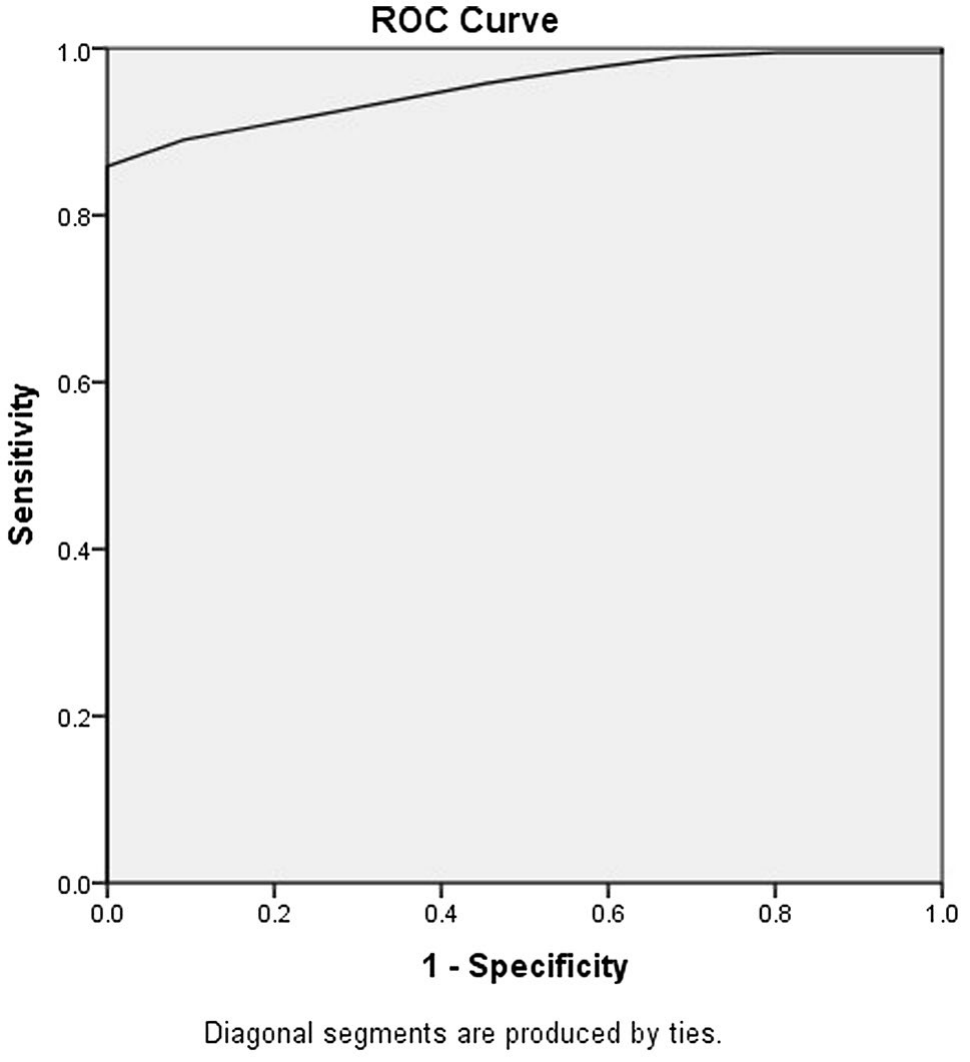
The ROC (receiver operating characteristic) curve between the preoperative radiological measure of PT depth and the intraoperative depth The area under the curve was 0.953

The sensitivity of our proposed method of measuring PT depth in the preoperative HRCT to predict the intraoperative PT depth was 91.71%. The specificity was 90.62%, the accuracy was 91.44%, the positive predictive value was 96.72%, the negative predictive value was 78.38%, and the Youden index was 0.82. At the same time, the ROC between the preoperative PT depth measurement and the intraoperative PT depth showed a strong correlation as the area under the curve was 0.953 (Fig. 2).

## Discussion

Posterior tympanotomy depth represented the thickness of the medial extension of the posterosuperior wall of the EAC. Most of the facial recess's previous anatomical or radiological analyses concentrated mainly on the width or the length dimensions. Only a few previous studies evaluated the depth dimension of the facial recess. Stuermer et al. measured the intraoperative thickness of the lateral part of the posterosuperior wall of the EAC. It ranged from 0.5 to 2.5 mm with a mean of 1.2 mm [11]. This measure did not represent the actual FR thickness as the bone becomes thicker medially. On the other hand, Wang et al. made a study on 16 adult cadaveric heads to evaluate the dimensions of the facial recess. In his anatomical study, the FR depth was 3.51 ± 0.17 mm [12].

HRCT has become a must in most centers before any cochlear implant surgery. It would help the surgeon analyze the temporal bone to detect anatomical variations and predict surgical difficulty. This would help efficient preparation and improve the cochlear implant outcomes [13].

Our radiological measure of the PT depth was a novel maneuver. We used the oblique para-sagittal plane. We tried to use fixed landmarks during our assessments to make a reproducible, straightforward method. Therefore, we used the plane parallel to the tip of the incus short process and the plane parallel to the medial extension of the posterosuperior wall of the EAC in the parasagittal cut of the HRCT. This measurement showed that the bone has to be drilled during posterior tympanotomy to access the middle ear. The reproducibility of our radiological method was confirmed by the statistically high agreement between both CT reviewers.

We correlated the preoperative radiological PT depth with intraoperative depth to validate this method. This qualitative correlation confirmed the ability of the radiological process to differentiate between the intraoperative ordinary PT depth and the deep posterior tympanotomy. This method's high sensitivity, specificity, and accuracy indicated its reliability in evaluating the PT depth.

On the other hand, our results revealed a close positive relationship between the PT depth and the intraoperative PT difficulty. When the depth increased, the PT became more difficult. This relation would help in the preoperative prediction of PT difficulty.

## Conclusions

We created a novel radiological method to measure the posterior tympanotomy depth. This method was valid, reproducible, and reliable in the preoperative radiological evaluation of the PT depth with high sensitivity (91.71%), specificity (90.62%), and accuracy (91.44%).
